# Burden and Associated Factors of Anaemia Among Rural School-Going Adolescent Girls in North Coastal Andhra Pradesh, India: A Cross-Sectional Study

**DOI:** 10.7759/cureus.111649

**Published:** 2026-06-28

**Authors:** Sagarika Duggirala, Saptarishi Bose, Revathi D Karuturi

**Affiliations:** 1 Community Medicine, Great Eastern Medical School and Hospital, Srikakulam, IND; 2 Child Health, Sudhir Heart Center, Berhampur, IND

**Keywords:** adolescent girls, anaemia, associated factors, india, iron deficiency, menstrual health, prevalence, rural population

## Abstract

Background

Anaemia remains a major public health problem among adolescent girls, particularly in low- and middle-income countries. Adolescence is associated with increased iron requirements due to rapid growth and the onset of menarche. In India, anaemia prevalence remains high despite ongoing national nutrition programmes, and district-level data remain limited in several regions.

Methods

A school-based cross-sectional study was conducted among 278 adolescent girls aged 13-17 years in Srikakulam district, Andhra Pradesh. Data on socio-demographic, dietary, and menstrual factors were collected using a semi-structured questionnaire. Haemoglobin levels were measured using a point-of-care haemoglobinometer. For consistency across the study population, anaemia was defined as haemoglobin <12 g/dL using the World Health Organization (WHO) criteria for non-pregnant adolescent females. Bivariate analysis and multivariable logistic regression were performed.

Results

The mean age of participants was 15.1 ± 1.4 years, and the mean haemoglobin level was 10.9 ± 1.8 g/dL. The prevalence of anaemia was 66.9% (95% confidence interval (CI): 61.4%-72.4%). In multivariable analysis, inadequate iron intake (adjusted odds ratio (AOR): 3.0; 95% CI: 1.8-5.2), tea consumption with meals (AOR: 2.0; 95% CI: 1.2-3.6), low green leafy vegetable intake (AOR: 1.8; 95% CI: 1.1-3.0), lower socioeconomic status (AOR: 1.6; 95% CI: 1.0-2.7), prolonged menstrual flow (AOR: 2.0; 95% CI: 1.2-3.4), and irregular menstrual cycles (AOR: 1.5; 95% CI: 1.0-2.8) were independently associated with anaemia.

Conclusions

Anaemia was highly prevalent among rural school-going adolescent girls in this study population. Inadequate iron intake, tea consumption with meals, low green leafy vegetable intake, lower socioeconomic status, prolonged menstrual flow, and irregular menstrual cycles were independently associated with anaemia. These findings highlight the multifactorial nature of anaemia and suggest that school-based programmes should address dietary practices, behavioural factors, and menstrual health in an integrated manner. Such approaches may contribute to reducing the burden of anaemia among adolescent girls.

## Introduction

Anaemia continues to represent a substantial nutritional and public health challenge among adolescent girls, particularly in resource-constrained settings [1]. Adolescence is a critical period of rapid growth and increased iron requirements, especially following menarche, which increases vulnerability to iron deficiency [2,3].

In India, anaemia among adolescent girls continues to remain high, with national surveys reporting prevalence exceeding 50% [4]. Despite large-scale initiatives such as the Weekly Iron and Folic Acid Supplementation programme and Anaemia Mukt Bharat, reductions in anaemia burden have been modest [5,6]. Anaemia during adolescence has significant health consequences, including impaired physical growth, reduced cognitive performance, decreased academic productivity, and adverse reproductive outcomes in later life [7-9].

The aetiology of anaemia in adolescent girls is multifactorial, involving inadequate dietary intake, poor iron bioavailability, menstrual blood loss, and underlying socioeconomic determinants [10-13]. Behavioural practices such as tea consumption with meals and low dietary diversity further contribute to impaired iron absorption and nutritional deficiencies.

In our previously published systematic review of anaemia among school-going adolescent girls in India [7], prevalence estimates ranged from approximately 40% to over 70%, highlighting substantial regional variability related to dietary practices, socioeconomic conditions, and programme implementation. However, district-level epidemiological data from regions such as North Coastal Andhra Pradesh remain limited, particularly with respect to integrated assessment of dietary, socioeconomic, and menstrual determinants.

The primary objective of this study was to estimate the prevalence and severity of anaemia among rural school-going adolescent girls in Srikakulam district. The secondary objective was to evaluate socio-demographic, dietary, and menstrual factors associated with anaemia.

## Materials and methods

Study design and setting

A school-based cross-sectional study was conducted to estimate the prevalence of anaemia and its association with selected socio-demographic, dietary, and menstrual factors. Cross-sectional designs are appropriate for estimating prevalence and studying associations at a single point in time [14].

The study was carried out in Srikakulam district, located in the North Coastal region of Andhra Pradesh, India. The district is predominantly rural, with significant socioeconomic and nutritional vulnerability. A total of 14 schools were included, comprising 10 Government Kasturba Gandhi Balika Vidyalaya (KGBV) schools and four private schools, enabling representation across different socioeconomic strata.

Study population and period

The study population consisted of school-going adolescent girls aged 13-17 years enrolled in the selected schools during the study period. The study was conducted over 18 months, from July 2024 to December 2025.

Inclusion criteria

School-going adolescent girls aged 13-17 years who were present on the day of data collection and provided assent, along with parental or guardian consent, were included in the study.

Exclusion criteria

Participants who were seriously ill at the time of data collection, had a known history of chronic haematological disorders or declined participation were excluded from the study.

Sample size estimation

The sample size was estimated using the standard formula for prevalence studies:

$n = \frac{Z^2 \times P \times q}{d^2}$

where *Z* = 1.96 for a 95% confidence level, *P* = anticipated prevalence, *q* = 1 − *P*, and *d* = allowable error. Based on previous reports indicating an anaemia prevalence of approximately 60%-70% among adolescent girls, the formula was used as a framework for sample-size planning. Considering feasibility, available resources, and the school-based nature of the study, a target sample size of approximately 260-300 participants was planned. A total of 278 participants were included in the final analysis [15].

Sampling technique

A school-based enrolment approach was adopted using convenience sampling within selected schools. Schools were selected to include both government and private institutions to ensure representation across different socioeconomic strata. Within each school, all eligible participants present on the day of data collection were invited to participate until the required sample size was achieved. Although convenience sampling was used, inclusion of multiple schools across socioeconomic groups was intended to improve the representativeness of the study population. However, the sampling approach may limit generalizability, and the findings should be interpreted with caution when extrapolating to the wider rural adolescent population of the district.

Data collection tool

Data were collected using a single semi-structured questionnaire developed by the investigators based on relevant literature and national guidelines, and pre-tested before field use. The instrument included sections addressing socio-demographic characteristics, dietary practices, menstrual history, and selected health-related variables. Dietary assessment formed one component of this questionnaire. The questionnaire was initially prepared in English, translated into Telugu, and subsequently back-translated into English to ensure consistency and semantic equivalence. The questionnaire is provided in the Appendix.

Dietary assessment

Dietary practices were assessed using the dietary component of the semi-structured questionnaire, focusing on the type of diet (vegetarian or mixed), frequency of consumption of green leafy vegetables, intake of iron-rich foods, and consumption of tea with meals. This assessment was based on self-reported frequency patterns rather than a quantitative dietary recall or a validated food frequency questionnaire.

Iron intake was categorised as adequate or inadequate based on the frequency of consumption of iron-rich foods in relation to recommended dietary practices. This simplified approach was adopted to capture key dietary behaviours relevant to iron intake in a field setting.

Menstrual history

Information regarding menstrual characteristics was collected from participants who had attained menarche, including age at menarche, duration of menstrual flow, and regularity of menstrual cycles.

Haemoglobin estimation

Haemoglobin concentration was measured using the Mission® Hb Haemoglobin Testing System (ACON Biotech (Hangzhou) Co., Ltd., Hangzhou, China). Capillary blood samples were obtained by finger prick under aseptic precautions and analysed according to the manufacturer's instructions. Point-of-care haemoglobin testing systems have been shown to provide acceptable accuracy and utility for anaemia screening in field and resource-constrained settings [16,17].

Operational definitions

According to World Health Organization (WHO) criteria, anaemia among non-pregnant adolescent girls was defined as a haemoglobin concentration below 12.0 g/dL [2]. Severity of anaemia was classified as mild (11.0-11.9 g/dL), moderate (8.0-10.9 g/dL), and severe (<8.0 g/dL) based on WHO recommendations [2]. WHO haemoglobin thresholds for adolescent girls and non-pregnant females were applied uniformly across the study population to maintain consistency of classification.

Socioeconomic status was assessed using the Modified B.G. Prasad classification updated for the Consumer Price Index and grouped into lower, middle, and upper categories [18].

Statistical analysis

Data were entered into Microsoft Excel and analysed using SPSS version 25 (IBM Corp., Armonk, NY). Descriptive statistics were used to summarise variables.

Associations between anaemia and independent variables were assessed using the chi-square test. Variables showing statistical significance in bivariate analysis, along with those of clinical relevance, were included in multivariable logistic regression analysis. Adjusted odds ratios with 95% confidence intervals (CIs) were reported. A *P*-value <0.05 was considered statistically significant.

Multicollinearity among independent variables was assessed using variance inflation factors (VIF). Model fit was evaluated using the Hosmer-Lemeshow goodness-of-fit test. Pseudo R² statistics were not the primary focus of analysis, as the objective was to identify significant associations rather than to develop a predictive model. Missing data, if any, were minimal and handled using complete-case analysis.

Ethical considerations

Ethical clearance was obtained from the Institutional Ethics Committee before commencement of the study. Permission was obtained from the education authorities and school heads. Written informed consent was obtained from parents or guardians, and assent was obtained from participants. Confidentiality of participant information was maintained throughout the study. Participants identified as anaemic were counselled and referred for appropriate management.

## Results

Baseline characteristics of study participants

A total of 278 adolescent girls were included in the analysis. The mean age of participants was 15.1 ± 1.4 years, and the mean haemoglobin level was 10.9 ± 1.8 g/dL. Overall, 186 participants (66.9%; 95% CI: 61.4%-72.4%) were anaemic, while 92 (33.1%) were non-anaemic.

Table 1 shows the baseline socio-demographic, dietary, and menstrual characteristics of the study population.

**Table 1:** Baseline characteristics of study participants (n = 278). Values are presented as mean ± standard deviation or number (%). *Menstrual variables are reported among participants who attained menarche. GLV, green leafy vegetable

Variable	Category	*n* (%)
Age (years)	Mean ± SD	15.1 ± 1.4
Haemoglobin (g/dL)	Mean ± SD	10.9 ± 1.8
Anaemia status	Non-anaemic	92 (33.1)
	Anaemic	186 (66.9)
School type	Government	198 (71.2)
	Private	80 (28.8)
Socioeconomic status	Lower	162 (58.3)
	Middle	96 (34.5)
	Upper	20 (7.2)
Diet type	Vegetarian	132 (47.5)
	Mixed	146 (52.5)
GLV intake	Regular	102 (36.7)
	Rare/Occasional	176 (63.3)
Iron intake	Adequate	98 (35.3)
	Inadequate	180 (64.7)
Tea with meals	No	132 (47.5)
	Yes	146 (52.5)
Menarche attained	No	26 (9.4)
	Yes	252 (90.6)
Menstrual flow*	≤5 days	140 (55.6)
	>5 days	112 (44.4)
Menstrual cycle*	Regular	164 (65.1)
	Irregular	88 (34.9)

The majority of participants attended government schools (198, 71.2%), while 80 (28.8%) attended private schools. More than half of the participants belonged to the lower socioeconomic group (162, 58.3%).

Dietary assessment revealed that 180 participants (64.7%) had inadequate iron intake, while 176 participants (63.3%) reported rare or occasional consumption of green leafy vegetables. Tea consumption with meals was reported by 146 participants (52.5%).

Menarche had been attained by 252 participants (90.6%). Among them, 112 participants (44.4%) reported prolonged menstrual flow (>5 days), and 88 participants (34.9%) reported irregular menstrual cycles.

Distribution of anaemia severity

**Figure 1:**
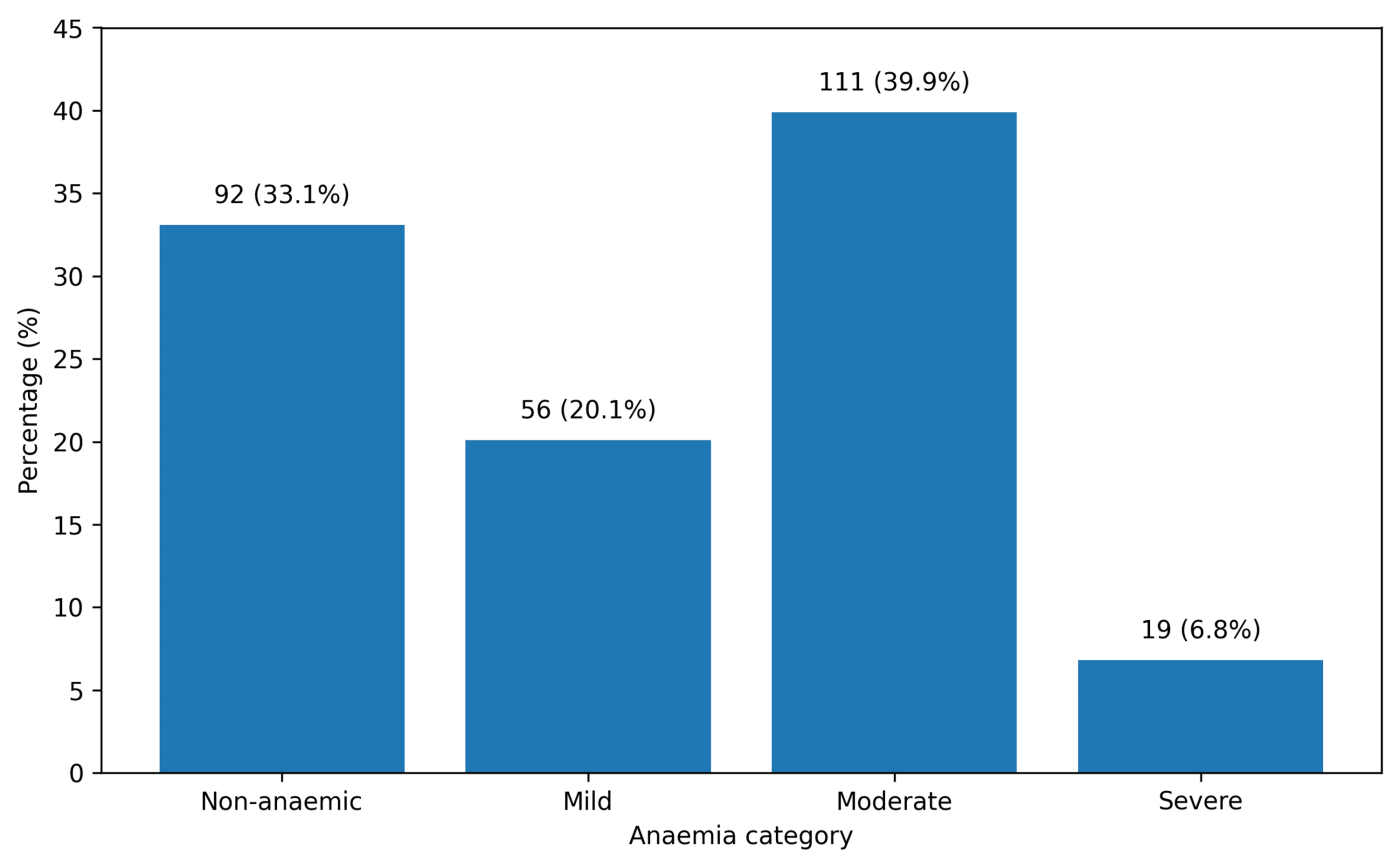
Distribution of anaemia severity among study participants (n = 278). Bars represent the percentage distribution of anaemia severity categories. Values above each bar indicate the corresponding frequency (n) and percentage (%). Anaemia severity was classified according to the World Health Organization (WHO) criteria.

The distribution of anaemia severity among study participants is presented in Figure 1.

Moderate anaemia was the most common category, affecting 111 participants (39.9%), followed by mild anaemia in 56 participants (20.1%). Severe anaemia was observed in 19 participants (6.8%), while 92 participants (33.1%) were non-anaemic.

Bivariate analysis of factors associated with anaemia

Table 2 presents the bivariate analysis of factors associated with anaemia.

**Table 2:** Bivariate analysis of factors associated with anaemia among study participants (n = 278). Values are presented as number (*n*) and percentage (%). *P*-values were calculated using the chi-square test. A *P*-value < 0.05 was considered statistically significant. *Menstrual flow duration and menstrual cycle characteristics were analysed among participants who had attained menarche. GLV, green leafy vegetable; SES, socioeconomic status

Variable	Category	Anaemic, *n* (%)	Non-anaemic, *n* (%)	*P*-value
Iron intake	Inadequate	150 (83.3)	30 (16.7)	<0.001
	Adequate	36 (36.7)	62 (63.3)	
Tea with meals	Yes	110 (75.3)	36 (24.7)	0.004
	No	76 (57.6)	56 (42.4)	
GLV intake	Rare	132 (75.0)	44 (25.0)	0.01
	Regular	54 (52.9)	48 (47.1)	
SES	Lower	122 (75.3)	40 (24.7)	0.03
	Middle/Upper	64 (55.2)	52 (44.8)	
Flow duration*	>5 days	88 (78.6)	24 (21.4)	0.02
	≤5 days	82 (58.6)	58 (41.4)	
Menstrual cycle*	Irregular	70 (79.5)	18 (20.5)	0.03
	Regular	101 (61.6)	63 (38.4)	

Anaemia prevalence was significantly higher among participants with inadequate iron intake (150, 83.3%) compared to those with adequate intake (36, 36.7%) (*P* < 0.001). Participants who consumed tea with meals had a higher prevalence of anaemia (110, 75.3%) compared to those who did not (76, 57.6%) (*P* = 0.004). Similarly, anaemia was more common among participants with low green leafy vegetable intake (132, 75.0%) compared to those with regular intake (54, 52.9%) (*P* = 0.01).

Socioeconomic status was significantly associated with anaemia, with a higher prevalence observed among participants from lower socioeconomic groups (122, 75.3%) compared to middle and upper groups (64, 55.2%) (*P* = 0.03). Among menstrual factors, prolonged menstrual flow (>5 days) was associated with a higher prevalence of anaemia (88, 78.6%) compared with a flow duration of ≤5 days (82, 58.6%) (*P* = 0.02). Irregular menstrual cycles were also significantly associated with anaemia (70, 79.5%) compared to regular cycles (101, 61.6%) (*P* = 0.03).

Multivariable logistic regression analysis

Table 3 shows the results of multivariable logistic regression analysis identifying independent predictors of anaemia.

**Table 3:** Multivariable logistic regression analysis identifying independent predictors of anaemia among study participants (n = 278). Reference categories were adequate iron intake, no tea consumption with meals, regular GLV intake, middle/upper SES, menstrual flow ≤5 days, and regular menstrual cycles. *P*-values < 0.05 were considered statistically significant. AOR, adjusted odds ratio; CI, confidence interval; GLV, green leafy vegetable; SES, socioeconomic status

Variable	Category	AOR	95% CI	*P*-value
Iron intake	Inadequate vs. adequate	3.0	1.8-5.2	<0.001
Tea consumption with meals	Yes vs. no	2.0	1.2-3.6	0.006
Green leafy vegetable intake	Low vs. regular	1.8	1.1-3.0	0.02
Socioeconomic status	Lower vs middle/upper	1.6	1.0-2.7	0.04
Menstrual flow (>5 days)	Yes vs. no	2.0	1.2-3.4	0.01
Menstrual cycle (irregular)	Yes vs. no	1.5	1.0-2.8	0.04

Inadequate iron intake emerged as the strongest independent predictor of anaemia, with participants having threefold higher odds of anaemia compared to those with adequate iron intake (AOR: 3.0; 95% CI: 1.8-5.2; *P* < 0.001). Tea consumption with meals was independently associated with anaemia, with approximately twofold higher odds (AOR: 2.0; 95% CI: 1.2-3.6; *P* = 0.006). Low intake of green leafy vegetables also remained a significant predictor (AOR: 1.8; 95% CI: 1.1-3.0; *P* = 0.02).

Participants from lower socioeconomic groups had higher odds of anaemia compared to those from middle and upper socioeconomic groups (AOR: 1.6; 95% CI: 1.0-2.7; *P* = 0.04). Among menstrual factors, prolonged menstrual flow (>5 days) (AOR: 2.0; 95% CI: 1.2-3.4; *P* = 0.01) and irregular menstrual cycles (AOR: 1.5; 95% CI: 1.0-2.8; *P* = 0.04) were independently associated with anaemia. Age, school type, and dietary pattern (vegetarian vs. mixed) were not independently associated with anaemia after adjustment.

## Discussion

This study demonstrates a high prevalence of anaemia (66.9%) among school-going adolescent girls in a predominantly rural district of North Coastal Andhra Pradesh. The mean haemoglobin level of 10.9 ± 1.8 g/dL further reflects a substantial burden of anaemia in this population. These findings are consistent with previous Indian studies and systematic evidence reporting anaemia prevalence ranging between 40% and 70% among adolescent girls, indicating that anaemia continues to remain a significant public health challenge despite ongoing national programmes [19-22]. However, given the cross-sectional design of the study, these findings should be interpreted as associations rather than causal relationships.

Burden and severity of anaemia

The distribution of anaemia severity in this study showed that moderate anaemia constituted the largest proportion (40%), followed by mild anaemia (20%) and severe anaemia (7%). This pattern suggests that a considerable proportion of affected individuals fall within clinically significant categories that may impact physical growth, cognitive performance, and future reproductive health. Similar distributions have been reported in Indian school-based studies, where moderate anaemia predominates over severe forms [7].

Dietary determinants

Nutritional practices were the strongest correlates of anaemia observed in the present study. Inadequate iron intake was associated with a markedly higher prevalence of anaemia (150, 83.3% vs. 36, 36.7%) and remained the strongest independent predictor in multivariable analysis (AOR: 3.0; 95% CI: 1.8-5.2). This finding reinforces the importance of inadequate dietary iron intake as a contributor to anaemia among adolescents, as consistently reported in previous studies [11-13]. Tea consumption with meals was also independently associated with anaemia (AOR: 2.0; 95% CI: 1.2-3.6). This association is biologically plausible, as polyphenols present in tea inhibit non-heme iron absorption and reduce iron bioavailability [11-13]. Given the high prevalence of tea consumption (146, 52.5%) in this study population, this represents an important modifiable behavioural risk factor.

Low intake of green leafy vegetables was significantly associated with anaemia both in bivariate and multivariable analyses (AOR: 1.8; 95% CI: 1.1-3.0). This reflects poor dietary diversity and inadequate micronutrient intake, which are well-established contributors to anaemia in adolescent populations [23,24].

Socioeconomic determinants

Socioeconomic status showed a clear gradient, with higher prevalence of anaemia among participants from lower socioeconomic groups (122, 75.3% vs. 64, 55.2%). This association persisted in multivariable analysis (AOR: 1.6; 95% CI: 1.0-2.7), indicating an independent association.

The observed association may reflect broader social and economic disadvantages that influence dietary quality and access to health services. Similar associations between low socioeconomic status and anaemia have been reported in previous studies from low- and middle-income settings [23,24].

Menstrual factors

Menstrual characteristics were also significantly associated with anaemia. Prolonged menstrual flow (>5 days) was associated with higher prevalence of anaemia (88, 78.6%) and remained an independent predictor (AOR: 2.0; 95% CI: 1.2-3.4). Similarly, irregular menstrual cycles were associated with increased odds of anaemia (AOR: 1.5; 95% CI: 1.0-2.8).

These findings are consistent with physiological mechanisms of increased iron loss during menstruation and higher iron requirements during adolescence [3]. The high proportion of participants who had attained menarche (252, 90.6%) underscores the importance of incorporating menstrual health into anaemia prevention strategies.

Non-significant factors

Age, school type, and overall dietary pattern (vegetarian versus mixed) were not independently associated with anaemia after adjustment. This suggests that specific dietary behaviours and nutrient intake patterns may be more relevant than broad dietary classifications.

Programmatic implications

Despite national initiatives such as the Weekly Iron and Folic Acid Supplementation programme and Anaemia Mukt Bharat, the high prevalence observed in this study suggests that programme coverage alone may not necessarily translate into improved haemoglobin outcomes [5,6]. Factors such as adherence, dietary practices and behavioural determinants may influence programme effectiveness.

Public health implications

These findings suggest that reducing anaemia among adolescent girls requires interventions that extend beyond iron supplementation alone. Given the influence of dietary, behavioural, socioeconomic, and menstrual factors identified in this study, school-based programmes should adopt a broader and more integrated approach. Efforts should focus on improving consumption of iron-rich foods, reducing tea consumption with meals to enhance iron absorption, promoting greater dietary diversity through regular intake of green leafy vegetables, and strengthening menstrual health awareness. Addressing these interconnected determinants through coordinated health and nutrition initiatives may contribute to more effective and sustainable reductions in the burden of anaemia among adolescent girls.

Strengths

This study provides primary field-based data from a region with limited published epidemiological evidence. The inclusion of both government and private schools enabled representation across socioeconomic strata. The use of a validated point-of-care haemoglobinometer ensured reliable measurement, and multivariable analysis allowed identification of independent predictors.

Limitations

The cross-sectional design limits causal inference, and the findings represent associations rather than causation. The study was conducted in a single district using convenience sampling, which may limit generalizability to other populations. Because a convenience sampling approach was used, the observed anaemia prevalence should be interpreted as representative of the study sample rather than a precise population estimate for all adolescent girls in the district. Dietary practices and menstrual history were self-reported, introducing the possibility of recall bias.

The dietary assessment was based on self-reported frequency patterns of food intake and selected dietary behaviours rather than a quantitative dietary recall, validated food frequency questionnaire, or detailed nutritional composition analysis of school meal programmes. Consequently, dietary adequacy and compliance with dietary reference intakes for iron and other micronutrients could not be directly assessed.

Important potential confounders such as helminthic infestation, malaria, micronutrient deficiencies other than iron (including vitamin B12 and folate), body mass index and overall nutritional status were not analysed, and adherence to iron supplementation programmes was not assessed. The omission of these variables may have resulted in residual confounding and could have influenced the observed associations. Furthermore, biochemical markers of iron status, such as serum ferritin or soluble transferrin receptor levels, were not measured. Therefore, the findings relating to inadequate iron intake should not be interpreted as evidence of laboratory-confirmed iron deficiency.

Additionally, WHO haemoglobin thresholds were applied uniformly across the adolescent age range to maintain consistency of classification; however, this approach may have resulted in minor classification differences across some age groups within the adolescent population. Tea consumption with meals was assessed using a simplified questionnaire-based approach, and detailed quantification of intake was not performed. The extended data-collection period may also have introduced temporal or seasonal variation in dietary practices and haemoglobin status that was not specifically evaluated in the present study. Future studies incorporating these parameters would provide a more comprehensive understanding of the determinants of anaemia.

## Conclusions

Anaemia among rural school-going adolescent girls in this study population was highly prevalent, affecting nearly two-thirds of participants. The burden was predominantly moderate in severity, indicating a substantial proportion of adolescents at risk of adverse health outcomes.

Dietary factors, particularly inadequate iron intake, were independently associated with anaemia. Behavioural practices such as tea consumption with meals and low intake of green leafy vegetables, along with lower socioeconomic status and menstrual factors, were also independently associated with anaemia.

These findings highlight the multifactorial nature of anaemia among adolescent girls and underscore the need for comprehensive, school-based approaches that extend beyond iron supplementation alone. Potential strategies may include improving dietary practices, promoting behaviour change to enhance iron absorption and incorporating menstrual health education. Strengthening implementation and adherence to existing national programmes, together with context-specific public health interventions, may contribute to reducing the burden of anaemia in this population.

## Appendices

Appendix

**Table 4:** Study questionnaire.

Section	Item	Response Options
A. Sociodemographic Information	Age (years)	Numeric
	School Class/Grade	Class VIII / Class IX / Class X
	Birth Order of Participant	First / Second / Third or above
	Type of Family	Nuclear / Joint / Extended
	Total Number of Family Members	Numeric
	Paternal Education	Illiterate / Primary / Secondary / Higher Secondary / Graduate and above
	Maternal Education	Illiterate / Primary / Secondary / Higher Secondary / Graduate and above
	Occupation of Head of Household	Open response
	Socioeconomic Status (Modified B.G. Prasad Classification)	Upper / Middle / Lower
B. Dietary Information	Green Leafy Vegetable Consumption	Yes / No
	Frequency of Green Leafy Vegetable Intake	Daily / Alternate days / Twice a week / Once a week
	Other Vegetable Consumption	Yes / No
	Frequency of Other Vegetable Intake	Daily / Alternate days / Twice a week / Once a week
	Fruit Consumption	Yes / No
	Frequency of Fruit Intake	Daily / Alternate days / Twice a week / Once a week
	Milk Consumption	Yes / No
	Frequency of Milk Intake	Daily / Alternate days / Twice a week / Once a week
	Tea After Meals	Yes / No
	Frequency of Tea Intake	Daily / Alternate days / Twice a week / Once a week
	Junk Food Consumption	Yes / No
	Frequency of Junk Food Intake	Daily / Alternate days / Twice a week / Once a week
	Regular Iron and Folic Acid (IFA) Supplementation	Yes / No
C. Menstrual Information	Age at Menarche (years)	Numeric
	Menstrual Cycle Frequency	Normal (21–35 days) / Polymenorrhoea (<21 days)
	Duration of Menstrual Blood Flow	3 days / 4 days / 5 days / More than 5 days
	Menorrhagia	Present (>5 days and/or >3 pads per day and/or passage of blood clots) / Absent
	Dysmenorrhoea	Present / Absent
	Menstrual Cycle Regularity	Regular / Irregular
D. Anthropometric and Clinical Assessment	Height (cm)	Numeric
	Weight (kg)	Numeric
	Body Mass Index (BMI) (kg/m²)	Numeric
	Clinical Signs of Anaemia	Present / Absent
	Haemoglobin Concentration (g/dL)	Numeric

## References

[1] World Health Organization (2008). Worldwide Prevalence of Anaemia 1993-2005. https://www.who.int/publications/i/item/9789241596657.

[2] World Health Organization (2011). Haemoglobin Concentrations for the Diagnosis of Anaemia and Assessment of Severity. https://www.who.int/publications/i/item/WHO-NMH-NHD-MNM-11.1.

[3] World Health Organization (2014). Adolescent Nutrition: A Review of the Situation. https://iris.who.int/items/4185498f-7685-4054-90e1-0056c12c9e4d.

[4] International Institute for Population Sciences (IIPS) and ICF. 2021.National Family Health Survey (NFHS-5), 2019 2019 (2019). International Institute for Population Sciences (IIPS) and ICF. 2021. National Family Health Survey (NFHS-5). National Family Health Survey (NFHS-5.

[5] Kapil U, Kapil R, Gupta A (2019). National Iron Plus Initiative: current status and future strategy. Indian J Med Res.

[6] Ministry of Health and Family Welfare, Government of India (2018). Anaemia Mukt Bharat: Intensified National Iron Plus Initiative Strategy. New Delhi: MoHFW.

[7] Duggirala S, Bose S, Karuturi RD (2026). Anaemia among school-going adolescent girls in India: a systematic review of prevalence, predictors, and Prevention pathways. Cureus.

[8] Beard JL (2000). Iron requirements in adolescent females. J Nutr.

[9] Kassebaum NJ, Jasrasaria R, Naghavi M (2014). A systematic analysis of global anemia burden from 1990 to 2010. Blood.

[10] Brabin BJ, Hakimi M, Pelletier D (2001). An analysis of anemia and pregnancy-related maternal mortality. J Nutr.

[11] Thankachan P, Walczyk T, Muthayya S, Kurpad AV, Hurrell RF (2008). Iron absorption in young Indian women: the interaction of iron status with the influence of tea and ascorbic acid. Am J Clin Nutr.

[12] Hurrell R, Egli I (2010). Iron bioavailability and dietary reference values. Am J Clin Nutr.

[13] Reddy MB, Agbemafle I, Armah S (2022). Iron Bioavailability: Enhancers and Inhibitors. Nutritional Anemia. Nutrition and Health.

[14] Setia MS (2016). Methodology series module 3: cross-sectional studies. Indian J Dermatol.

[15] Lwanga SK, Lemeshow S (1991). Sample Size Determination in Health Studies: A Practical Manual. https://tbrieder.org/publications/books_english/lemeshow_samplesize.pdf.

[16] Sahoo J, Epari V, Panigrahi SK, Prasad D, Bhola RK, Mohanty S, Behera BK (2021). Challenges in detection of adolescent anaemia: validation of point-of-care device (Mission® plus) for haemoglobin measurement among tribal residential school children of selected districts of Odisha, India. Indian J Community Med.

[17] Brehm R, South A, George EC (2024). Use of point-of-care haemoglobin tests to diagnose childhood anaemia in low- and middle-income countries: A systematic review. Trop Med Int Health.

[18] Ghodke M (2023). Updated BG Prasad’s socioeconomic status classification for the year 2023. Indian J Community Med.

[19] Sahoo J, Mohanty S, Gupta S, Panigrahi SK, Mohanty S, Prasad D, Epari V (2023). Prevalence and risk factors of iron deficiency anemia among the tribal residential adolescent school students of Odisha: a cross-sectional study. Indian J Community Med.

[20] Chandrakumari AS, Sinha P, Singaravelu S, Jaikumar S (2019). Prevalence of anemia among adolescent girls in a rural area of Tamil Nadu, India. J Family Med Prim Care.

[21] Ahankari AS, Myles PR, Fogarty AW, Dixit JV, Tata LJ (2017). Prevalence of iron-deficiency anaemia and risk factors in 1010 adolescent girls from rural Maharashtra, India: a cross-sectional survey. Public Health.

[22] Kamble BD, Gunjan M, Sumit J, Singh SK, Jha D, Singh S (2021). Prevalence of anaemia among school going adolescent girls attending Test, Treat and Talk (T-3) camp under Anaemia Mukt Bharat in Delhi. J Family Med Prim Care.

[23] Balarajan Y, Ramakrishnan U, Ozaltin E, Shankar AH, Subramanian SV (2011). Anaemia in low-income and middle-income countries. Lancet.

[24] Chaparro CM, Suchdev PS (2019). Anemia epidemiology, pathophysiology, and etiology in low- and middle-income countries. Ann N Y Acad Sci.

